# A novel nuclear marker and development of an ARMS-PCR assay targeting the metallopeptidase 10 (*nas 10*) locus to identify the species of the *Anisakis simplex* (s. l.) complex (Nematoda, Anisakidae)

**DOI:** 10.1051/parasite/2020033

**Published:** 2020-05-26

**Authors:** Marialetizia Palomba, Michela Paoletti, Stephen C. Webb, Giuseppe Nascetti, Simonetta Mattiucci

**Affiliations:** 1 Department of Public Health and Infectious Diseases, Section of Parasitology, “Sapienza – University of Rome” Piazzale Aldo Moro 5 00185 Rome Italy; 2 Department of Ecological and Biological Sciences, Tuscia University, Viale dell’Università s/n 01100 Viterbo Italy; 3 Cawthron Institute 98 Halifax Street East Nelson 7010 New Zealand

**Keywords:** *Anisakis simplex* (s. s.), *A. pegreffii*, *A. berlandi*, Diagnostic markers, Metallopeptidase *nas 10* nDNA, ARMS-PCR

## Abstract

The genus *Anisakis* represents one of the most widespread groups of ascaridoid nematodes in the marine ecosystem. Three closely related taxa are recognized in the *Anisakis simplex* (s. l.) complex: *A. pegreffii*, *A. simplex* (s. s.) and *A. berlandi.* They are widely distributed in populations of their intermediate/paratenic hosts (fish and squids) and definitive hosts (cetaceans). A novel nuclear gene locus, metallopeptidase 10 (*nas 10*) (451 bp), was sequenced and validated on a total of 219 specimens of the three species of *Anisakis,* collected in fish and cetacean hosts from allopatric areas included in their ranges of distribution. The specimens of *Anisakis* were first identified by allozymes and sequence analysis of the mtDNA *cox2* and EF1*α*-1 nDNA. The novel nuclear marker has shown fixed alternative nucleotide positions in the three species, i.e. diagnostic at 100%, permitting the species determination of a large number of specimens analyzed in the present study. In addition, primers to be used for amplification-refractory mutation system (ARMS) PCR of the same gene locus were designed at these nucleotide positions. Thus, direct genotyping determination, by double ARMS, was developed and validated on 219 specimens belonging to the three species. Complete concordance was observed between the tetra-primer ARMS-PCR assays and direct sequencing results obtained for the *nas 10* gene locus. The novel nuclear diagnostic marker will be useful in future studies on a multi-locus genotyping approach and also to study possible hybridization and/or introgression events occurring between the three species in sympatric areas.

## Abstract (fr)

Le genre *Anisakis* représente l’un des groupes de nématodes ascaridoïdes les plus répandus dans l’écosystème marin. Trois taxons étroitement apparentés sont reconnus dans le complexe *Anisakis simplex* (s. l.) : *A. pegreffii*, *A. simplex* (s. s.) et *A. berlandi*. Ils sont largement répartis dans les populations de leurs hôtes intermédiaires/paraténiques (poissons et calmars) et définitifs (cétacés). Un nouveau locus de gène nucléaire, la métallopeptidase 10 (*nas 10*) (451 pb), a été séquencé et validé sur un total de 219 spécimens des trois espèces d’*Anisakis*, collectés chez des hôtes poissons et cétacés de zones allopatriques incluses dans leur aire de répartition. Les échantillons d’*Anisakis* ont d’abord été identifiés par des allozymes et une analyse des séquences de l’ADNmt *cox2* et de l’ADNn EF1α-1. Le nouveau marqueur nucléaire a montré des positions de nucléotides alternatives fixes dans les trois espèces, c’est-à-dire qu’il a permis un diagnostic à 100%, permettant la détermination de l’espèce d’un grand nombre d’échantillons analysés dans la présente étude. De plus, des amorces à utiliser pour la PCR par système de mutation réfractaire à l’amplification (ARMS) du même locus génique ont été conçues à ces positions nucléotidiques. Ainsi, la détermination directe du génotypage, par double ARMS, a été développée et validée sur 219 spécimens appartenant aux trois espèces. Une concordance complète a été observée entre les dosages ARMS PCR tétra-amorces et les résultats de séquençage direct obtenus pour le locus du gène *nas 10*. Le nouveau marqueur de diagnostic nucléaire sera utile dans les travaux futurs d’une approche de génotypage multi-locus et également pour étudier les éventuels événements d’hybridation et/ou d’introgression se produisant entre les trois espèces dans des zones sympatriques.

## Introduction

The *Anisakis simplex* (s. l.) species complex includes heteroxenous nematodes belonging to three distinct species, namely *A. pegreffii*, *A. simplex* (s. s.) and *A. berlandi* [25]. The three taxa, first genetically detected by allozymes [20], were later morphologically described [23], and recognized as distinct biological species based on different nuclear [33] and mitochondrial gene loci [35]. The geographical range of these three species is quite distinct; however, some oceanographic basin waters are reported as overlapping areas and contact zones between them (for a review, [25]). In allopatric and sympatric areas, they are known to infect several definitive and intermediate/paratenic hosts; the three species often being found parasitizing the same host-species range [25]. In addition, *A. pegreffii* and *A. simplex* (s. s.) are recognized as zoonotic parasites that cause human gastro- (GA) intestinal- (IA) anisakiasis and gastro-allergic (GAA) anisakiasis [25]. Gastro-intestinal anisakiasis is currently widespread in Europe, mostly occurring in Italy, Spain, France, Portugal and Croatia (reviewed in [9, 25]); cases have also been widely reported in Japan and South Korea [15]. Humans are accidental hosts who become infected after the ingestion of raw fish and squids containing, in their edible parts, the third-stage larvae (L3) of *A. pegreffii* and *A. simplex* (s. s.). No data are so far available about the zoonotic role of the species *A. berlandi*. On the other hand, while it was widely demonstrated that *A. simplex* (s. s.) and *A. pegreffii* [6, 13, 14] infect the musculature of the fish hosts, there is no knowledge about the ability of *A. berlandi* to invade the fish host musculature.

In recent years, the multi-locus genotyping approach has been suggested to identify nematodes of the genus *Anisakis* and to investigate certain micro-evolutionary aspects, such as hybridization and introgression phenomena [25]. With this aim, there have been several recent attempts to identify new nuclear markers. These approaches include: (1) sequence analysis of the nuclear locus EF1 *α*-1 nDNA showing the presence of fixed alternative nucleotide positions between the two species *A. pegreffii* and *A. simplex* (s. s.) [24], and (2) the development of DNA microsatellite loci [27]. According to the findings of these authors, no diagnostic markers were discovered between the two taxa [27]. On the other hand, opposite results were obtained by further recent development of polymorphic SSRs DNA markers [26]. Diagnostic SSRs DNA loci between the species *A. pegreffii* and *A. simplex* (s. s.) were detected [26], also with respect to the species *A. berlandi* [3], allowing the reliable identification of the species. It was also found that among the diagnostic loci detected at the SSRs loci between the three *Anisakis* species [3, 26], sex-linked loci exist. This last finding, however, highlighted the problem that, because male worms are hemizygotes at these diagnostic SSRs loci, their genotypes cannot be considered in a large genotyping analysis in the investigation of hybridization and introgression phenomena [26]. Moreover, it has also been underlined that a multi-locus nuclear genotyping approach is generally mandatory and highly recommended to investigate the occurrence of mixed ancestry only in the hosts and geographic areas where parasite species overlap in their range of distribution [1, 2, 7, 25]. Therefore, the discovery of further nuclear diagnostic markers to be used for disentangling hybridization and/or introgression events between these sibling species of *Anisakis* is highly desirable.

The amplification-refractory mutation system (ARMS) can distinguish a single base sequence difference using one-step PCR [32]. This method is based on the use of sequence-specific PCR primers that allow amplification of test DNA only when the target allele is contained within the sample. Following an ARMS reaction, the presence or absence of a PCR product is diagnostic for the presence or absence of the target allele. An ARMS-PCR assay was recently developed on ITS rDNA for the species identification of *A. pegreffii, A. simplex* (s. s.) and *A. typica* occurring in fish from Korean waters [11].

The scope of this work was (1) to provide a new nuclear diagnostic marker for the three *Anisakis* species (i.e. *A. pegreffii, A. simplex* (s. s.) and *A. berlandi*), based on the direct sequence analysis of a gene locus coding for a metallopeptidase 10 (*nas 10*), to be used in a future multi-locus approach; (2) to validate the novel marker on a large number of specimens of the three *Anisakis* species; and (3) to develop and validate an ARMS-PCR tool to genotype the diagnostic single nucleotide polymorphisms (SNPs) detected at *nas 10*, for rapid genotyping of individuals of *A. simplex* (s. s.), *A. pegreffii* and *A. berlandi*.

## Materials and methods

### Parasite sampling and molecular identification

A total of 219 *Anisaki*s spp. third-stage larvae and adults collected from intermediate/paratenic fish and cetacean hosts, respectively were obtained from the frozen and alcohol collection stored in the Section of Parasitology, Department of Public Health and Infectious Diseases of “Sapienza – University in Rome”. Details concerning the sampling localities are given in Table 1.

**Table 1:** Sampling area, host species and life-history stage (A: adult; L4: 4th stage larvae; L3: 3rd stage larvae) of the specimens of *A. simplex* (s. s.), *A. pegreffii* and *A. berlandi* analyzed.

Species	Sampling area	Host species	*N*	*N*_A_	*N*_L4_	*N*_L3_
*A. simplex* (s. s.)						
	NE Atlantic Ocean					
	Norwegian Sea	*Clupea harengus*	22	–	–	22
	(68°52′ N – 3°08′ E)	*Scomber scombrus*	2	–	–	2
	North Sea	*Clupea harengus*	3	–	–	3
	(59°13′ N – 00°14′ W)					
	Baltic Sea	*Clupea harengus*	9	–	–	9
	(58°29′ N – 19°51′ E)					
	English Channel	*Clupea harengus*	19	–	–	19
	(48°38′ N – 4°34′ W)					
	Gran Sole Bank	*Merluccius merluccius*	16	–	–	16
	(49°38′ N – 10°10′ W)					
	Scotland coast	*Stenella coeruleoalba*	13	13	–	–
		*Lagenorhynchus albirostris*	9	9	–	–
		*Total*	93	22	–	71
*A. pegreffii*						
	Mediterranean Sea					
	Tyrrhenian sea	*Merluccius merluccius*	1	–	–	1
	(41°7′ N – 13°24′ E)	*Lepidopus caudatus*	11	–	–	11
		*Scomber scombrus*	4	–	–	4
		*Trachurus trachurus*	1	–	–	1
	Western Adriatic Sea	*Stenella coeruleoalba*	22	11	1	10
	(42°18′ N – 15°35′ E)	*Lophius piscatorius*	1	–	–	1
		*Scomber scombrus*	5	–	–	5
	SW Pacific Ocean					
	New Zealand	*Globicephala melas*	31	20	11	–
	(44°30′ S – 172°58′ E)					
		*Total*	76	31	12	33
*A. berlandi*						
	SW Pacific Ocean					
	New Zealand	*Globicephala melas*	50	19	2	3
	(44°30′ S – 172°58′ E)					
		*Total*	50	19	2	3

The allozymes proven to be diagnostic for the species of the *A. simplex* (s. l.) complex were used [22, 23]. Standard horizontal starch gel electrophoresis was performed at these enzyme loci; their staining procedures are those previously reported [23]. Total DNA was extracted from a tissue portion (≈2 mg) of each *Anisakis* larva (L3, L4) and each adult specimen (Table 1). The *Quick*-gDNA^TM^ Miniprep Kit (ZYMO RESEARCH) was used as the extraction method. DNA obtained was quantified using a NanoDrop^®^TC1-E20 spectrophotometer (BioTek Synergy HT).

The specimens were identified to the species level by sequences analysis of the mtDNA *cox2* gene locus, which makes it possible to distinguish the three species *A. simplex* (s. s.), *A. pegreffii,* and *A. berlandi* [23]. Additionally, the specimens recognized at the mtDNA *cox2* locus as belonging to *A. pegreffii* and *A. simplex* (s. s.) were also identified by sequences analysis of EF1 *α*-1 nDNA [24].

For sequencing of the mtDNA *cox2* gene locus, PCR amplification was performed using the primers 211F (5′–TTT TCT AGT TAT ATA GAT TGR TTT YAT–3′) and 210R (5′–CAC CAA CTC TTA AAA TTA TC–3′) [23, 35]. PCR conditions were established as previously described [24]. The amplification of EF1 *α*-1 nDNA was performed using the primers EF-F (5′–TCC TCA AGC GTT GTT ATC TGT T–3′) and EF-R (5′–AGT TTT GCC ACT AGC GGT TCC–3′), under the conditions previously described [24].

### Primer selection for the *nas 10* nDNA and PCR condition

For direct sequencing of the metallopeptidase (*nas 10* nDNA) gene locus of *Anisakis*, oligonucleotide primers were newly designed based on the sequence at the metallopeptidase *asnas 10* of *Ascaris suum*, deposited in GenBank under accession number JI170268.1, by using the software program PRIMER3 plus (http://bioinfo.ut.ee/primer3-0.4.0/). The total sample of *N* = 219 individuals, belonging to the three studied species, previously identified by allozymes, mtDNA *cox2* and EF1 *α*-1 nDNA, was thus sequenced at the metallopeptidase 10 (*nas 10*) nDNA gene locus. Thus, PCR amplification was performed using the primers nas10-F (5′–GAT GTT CCT GCA AGT GAT TG–3′) and nas10-R (5′–CGC TAT TAA GAG AGG GAT CG–3′) (Table 2). PCR reactions were carried out in a 25 μL volume containing 0.6 μL of each primer 10 mm, 2 μL of MgCl_2_ 25 mm (Promega), 5 μL of 5× buffer (Promega), 0.6 μL of dNTPs 10 mm (Promega), 0.2 μL of Go-*Taq* Polymerase (5U/μL) (Promega) and 2 μL of total DNA. PCR temperature conditions were the following: 94 °C for 5 min (initial denaturation), followed by 40 cycles at 95 °C for 1 min (denaturation), 53 °C for 1 min (annealing), 72 °C for 1 min (extension), and post-amplification at 72 °C for 15 min.

**Table 2:** Primer sequences targeting the metallopeptidase 10 (*nas 10*) locus, product size, and annealing temperature (*T*_a_/°C). In bold, a deliberate second mismatch.

Name	Primer sequences (5′–3′)	Genotyping pattern (bp)	*T*_a_/°C
*nas 10*	F: GATGTTCCTGCAAGTGATTG	451 bp	53
	R: CGCTATTAAGAGAGGGATCG		
Primer set-1	Out-F1: TATGGCAAATATTATTATCGTA	373 bp (control fragment)	49
	Out-R1: TATTTCCGACAGCAAACAA	296 bp (T allele)	
	In-F1: GCATTGTACACTTCGTA**T**ATT	117 bp (C allele)	
	In-R1: ATTTCTYCAGCAATCGT**A**AG	373 bp (control fragment)	
Primer set-2	Out-F2: GAAAGACAGGTTCATCTCA	321 bp (control fragment)	49
	Out-R1: TATTTCCGACAGCAAACAA	117 bp (G allele)	
	In-F2: AACGGATATGAATGAT**C**CC	216 bp (C allele)	
	In-R2: HAAATGAAAGTAGAAAGAATT**T**AC	321 bp (control fragment)	

### Sequence data analysis

Clustal X (version 2.0) software [12] was used for sequence alignment, obtained at the studied gene loci (i.e. mtDNA *cox2*, EF1 *α*-1 nDNA and *nas 10* nDNA). In the case of mtDNA *cox2* and EF1 *α*-1 nDNA, the generated sequences were aligned with those from the same species previously obtained [22, 23], to detect the fixed diagnostic nucleotide positions that can be used to discriminate *A. simplex* (s. s.), *A. pegreffii* and *A. berlandi* [22, 23].

### Development of an ARMS-PCR assay targeting the *nas 10* nDNA locus

The diagnostic nucleotide positions found between the three *Anisakis* species were used to develop ARMS-PCR assay experiments. This resulted in the use of 4 pairs of primers organized into 2 primers sets (Table 2). The two sets were specifically designed for these diagnostic nucleotide positions (i.e. 173 and 251) observed at direct sequencing of *nas 10* nDNA, in the three species of the *A. simplex* (s. l.), by using the web-based program, Primers3 plus (https://primer3plus.com/primer3web/primer3web).

Each primer set includes four primers, one pair of primers comprises the forward and reverse outer primers which serve as internal amplification controls; the other pair of primers comprises the forward and reverse inner primers (Table 2). Importantly, in tetra-primer ARMS-PCR, there is not only a 3′ terminus mismatch but also a second deliberate mismatch at position-2 from the 3′ terminal of the inner primers to increase the specificity of the reaction. The two primer sets (Table 2) were used in two PCR reactions planned to work simultaneously, to identify *A. pegreffii*, *A. simplex* (s. s.) and *A. berlandi.* Each PCR was performed in a total volume of 25 μL: 3.5 μL of 10× solution buffer, 1.5 μL containing 10 μM of four mixed dNTPs, 1.5 μL of 50 Mm MgCl_2_, 1.2 μL of each primer (10 Mm) (Table 2), 0.25 μL of Taq DNA polymerase (5U/μL) (Promega) and 3 μL of total DNA. PCR amplification conditions were as follows: 95 °C for 5 min (initial denaturation) followed by 35 cycles at 95 °C for 30 s (denaturation), 49 °C for 35 s (annealing), 72 °C for 30 s (extension), and a final elongation step at 72 °C for 7 min. The PCR products generated from the outer primers were used as positive controls in the ARMS-PCR system to ensure the normality of PCR reaction (373 bp for primer set-1, 321 bp for primer set-2). The PCR products, obtained from each analyzed *Anisakis* specimen, by use of two sets of primers (Table 2), were separated by electrophoresis using agarose gel (1.5%) stained with GelRed^®^; 3 μL of the amplification products were visualized. The distinct banding patterns were detected by the use of ultraviolet transillumination.

## Results

### Identification of *Anisakis simplex* (s. l.) specimens

Allozyme analysis of *Anisakis* (*N* = 219 specimens) (Table 1) showed that 93 specimens were *A simplex* (s. s.), 50 *A. berlandi*, and finally 76 *A. pegreffii*, according to alleles found at the diagnostic loci between the three sibling species (i.e. *Adk-2*, *Pep C-1, Pep C-2,* and *Mdh-1*) [22]. Similarly, according to the sequences of 629 bp in length of the mtDNA *cox2* gene locus [22], 76 specimens were assigned to *A. pegreffii*, 93 to the species *A. simplex* (s. s.) and, finally, 50 individuals corresponded to *A. berlandi.* The sequences obtained in the present study matched, at 100% or 99%, with the sequences previously deposited by us in GenBank for these species. In addition, the identity of these specimens recognized at mtDNA *cox2* as belonging to *A. pegreffii* and *A. simplex* (s. s.), was confirmed according to the diagnostic positions of the EF1 *α*-1 nDNA locus [23].

### Genotyping of *Anisakis* spp. by direct sequencing of *nas 10* nDNA

**Figure 1:**
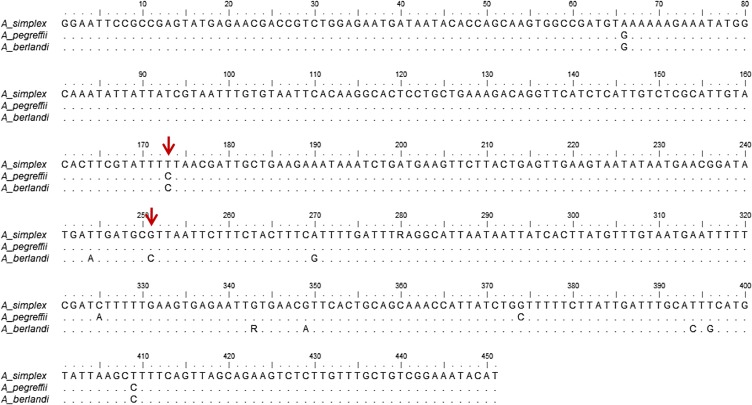
Sequence alignment of the nucleotide sequences of the metallopeptidase 10 (*nas 10*) of *A. simplex* (s. s.), *A. pegreffii* and *A. berlandi*. Dots indicate identity with the consensus sequence. Arrows indicate the nucleotide diagnostic position.

A fragment of 451 bp in length of the *nas 10* nDNA region was obtained for all of the 219 specimens analyzed here. The sequences revealed the presence of one diagnostic nucleotide site between *A. simplex* (s. s.) *versus A. pegreffii/A. berlandi*, and another one between *A. berlandi versus A. pegreffii/A. simplex* (s. s.): these diagnostic nucleotide positions were 173 and 251, respectively (Figure 1). In particular, position 173 showed the T allele in all the *A. simplex* (s. s.) individuals (*N* = 93); while it always showed the C allele in the specimens of *A. pegreffii* and *A. berlandi* considered here (Fig. 1). Position 251 showed the G allele in the individuals of *A. pegreffii* and *A. simplex* (s. s.), while the allele was always C in all the *A. berlandi* specimens (*N* = 50) analyzed here (Fig. 1). All the specimens belonging to the three *Anisakis* species were found to be homozygous at these nucleotide positions; no heterozygosity between the three species was found in the present study, at these positions. As a consequence, since these nucleotide positions are fixed for alternative nucleotides among the three species, they were considered to have diagnostic value. Sequences of the *nas 10* nDNA region were deposited in GenBank under the following accession numbers: MN897674, MN897675, MN897676 for *A. pegreffii*, MN897671, MN897672, MN897673 for *A. simplex* (s. s.), and MN897677, MN897678, MN897679 for *A. berlandi*.

### Genotyping of *Anisakis* spp. by ARMS-PCR targeting *nas 10* nDNA

The two point mutations diagnostic between the three *Anisakis* species and the tetra-primer ARMS-PCR analysis enabled us to genotype the individuals belonging to the three species studied here. All the specimens belonging to the three taxa, previously identified by allozymes, mtDNA *cox2*, and EF1 *α*-1 nDNA and sequenced at the *nas 10* nDNA locus, were directly analyzed by ARMS-PCR at this locus.

**Figure 2:**
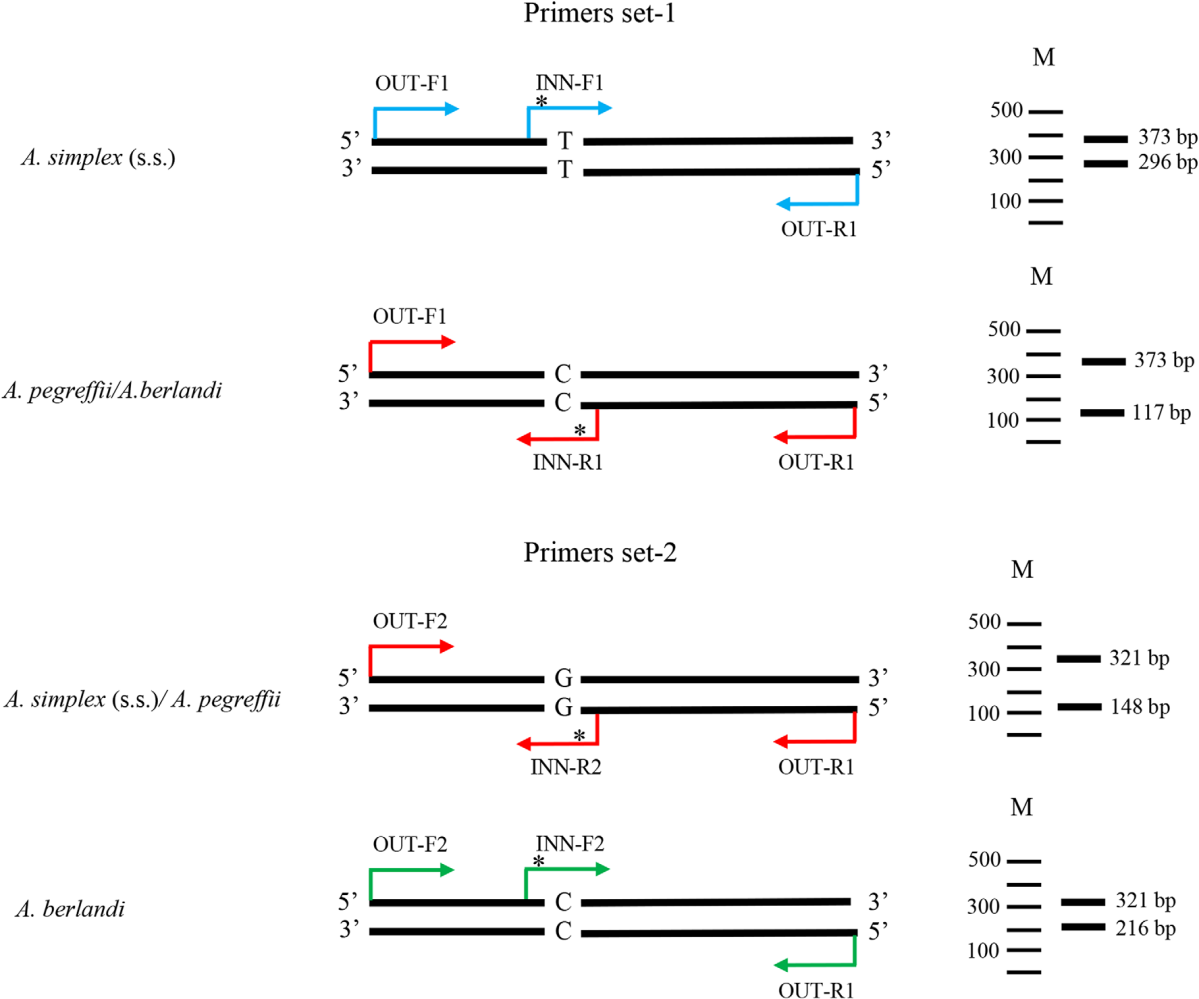
Schematic illustration of the two sets of the tetra-primers for the ARMS-PCR design and DNA gel patterns of the different single nucleotide genotyping (on the right). Asterisks indicate the second mismatch of the inner primer.

One set of designed primers (primer set-1) (Table 2) was able to detect the first point mutation (i.e. 173) making it possible to distinguish *A. simplex* (s. s.) *versus A. pegreffii/A. berlandi*; while, the second set of primers detected the point mutation 251, which always identified a C/C genotype in *A. berlandi*; while it showed a G/G genotype in both *A. simplex* (s. s.) and *A. pegreffii*. In particular, in primer set-1 (Table 2) the combined use of the *nas 10*-reverse outer primer (OUT-R1) and *nas 10*-forward inner primer (INN-F1), amplifying the T-allele at position 173, generated a specific PCR product of 296 bp in *A. simplex* (s. s.) (Fig. 2; Table 3). Similarly, the combined use of *nas 10*-forward outer primer (OUT-F1) and *nas 10*-reverse inner primer (INN-R1), amplifying the C-allele at position 173, generated a specific PCR fragment of 117 bp in *A. pegreffii* and *A. berlandi* specimens (Fig. 2; Table 3). In primer set-2 (Table 2), the combined *nas 10*-forward outer primer (OUT-F2) and *nas 10*-reverse inner primer (INN-R2) amplified the G-allele at the diagnostic nucleotide position 251, producing a specific PCR fragment of 148 bp in *A. simplex* (s. s.) and *A. pegreffii* (Fig. 2; Table 3). In the same primer set, the *nas 10*-forward inner primer (INN-F2) and the *nas 10*-reverse outer primer (OUT-R1) amplified the C-allele (diagnostic position 251) and determined a PCR product of 216 bp in *A. berlandi* (Fig. 2; Table 3).

**Table 3:** Molecular key based on the diagnostic positions.

Position (bp)	Genotype		Species identification	ARMS-PCR fragment pattern (bp)
**(1.)** 173	T/T	→	*A. pegreffii*/*A. simplex* (s. s.)	373 and 296
	C/C	→	*A. pegreffii*/*A. berlandi* **(2.)**	373 and 117
**(2.)** 251	G/G	→	*A. pegreffii*	321 and 148
	C/C	→	*A. berlandi*	321 and 216

All the specimens were homozygous for these fixed alternative nucleotide positions; no heterozygosity at these positions was found in the samples of *Anisakis* spp. tested in this study.

**Figure 3:**
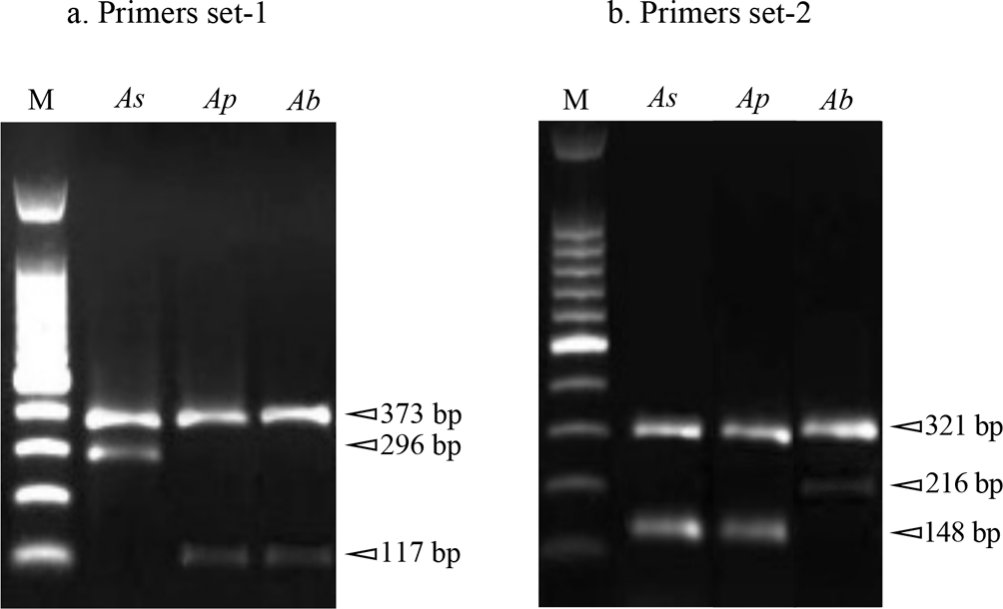
Products (genotypes) of tetra-primer ARMS-PCR obtained at the *nas 10* locus in the three species of the *A. simplex* (s. l.) complex.

On the basis of the gel electrophoresis patterns (Figure 3) obtained by the combined use of tetra-primer set-1 and tetra-primer set-2, 76 specimens were found to be *A. pegreffii*, 93 *A. simplex* (s. s.), and 50 *A. berlandi*. The findings showed 100% concordance between the genotyping obtained by the ARMS-PCR assay and direct sequence analysis of the same gene locus *nas 10* nDNA.

## Discussion

In parasitic nematodes, peptidase proteins represent around 2% of the genes expressed; they are involved in host-parasite interactions, parasite immune evasion, life cycle transmission, and pathogenesis [19]. In many cases, peptidases are also the main source of parasite antigens, favoring the host immune response; many of them are particularly immunogenic [10, 34]. In particular, metallopeptidases are a group of peptidases that are involved in the invasion of the host tissue by the parasite as they are able to degrade the extracellular matrix. In some cases, it has been suggested that the metallopeptidases conserve their proteolytic activity even after the antibody action exhibited by the host during infection by the parasite [4, 5, 18].

Recent RNA sequence analysis revealed a high percentage of the gene coding for metallopeptidases highly expressed in *A. pegreffii* third-stage larvae maintained *in vitro* co-cultured with dendritic cells, at 37 °C (Palomba et al. personal obs.). These enzymes were postulated to be involved in host tissue penetration by *Anisakis* spp. larvae [11]. The ability of the parasite to migrate to tissue would represent an evolutionary advantage for the larvae of *Anisakis* spp. during intermediate/paratenic host infection. In turn, this would mean that selective pressure would have conferred an evolutionary advantage to the genes coding for the group of peptidase proteins, allowing the parasite species to successfully invade different fish hosts. As a consequence, a high level of mutations would have occurred in these gene loci over an evolutionary time scale, resulting in an advantageous genotype for the parasite species, in terms of invasion and adaptation to its hosts’ ranges. Interestingly, previous allozyme studies in anisakids have demonstrated that among the scored gene loci, those coding for the peptidase proteins represent a high percentage showing fixed alternative alleles between related anisakid species belonging to the genera *Anisakis*, *Pseudoterranova* and *Contracaecum* [22, 28, 30, 31]. Furthermore, in the case of the three species of the *A. simplex* (s. l.) complex, the gene locus coding for the enzymatic proteins: Peptidase-C, Peptidase-B, and Leucine-amino-peptidase (i.e. coding loci: *Pep-C-1, PepC-2, Pep B,* and *Lap-1,* respectively) showed fixed alternative alleles that are diagnostic in these species. Thus, it seems that these gene loci coding for peptidases are fast evolving loci in anisakids [21], suitable for discriminating between closely related taxa [8].

The possibility of looking directly at the gene sequences coding for these functional proteins, such as peptidases, enables detection of fixed nucleotide positions. These positions are associate with a polymorphic sequence caused by a single nucleotide mutation at a specific locus in the DNA sequence. This type of polymorphism was found through direct sequencing of the gene coding for the metallopeptidase 10 (*nas 10* nDNA) presented, which showed alternative single nucleotide positions among the three *Anisakis* species: i.e. a further diagnostic nuclear gene. A molecular key based on these nucleotide differences is presented in Table 3. No heterozygous genotypes were observed: this is another demonstration that *A. pegreffii*, *A. simplex* (s. s.) and *A. berlandi* are independent gene pools.

The presence of the fixed differences can be considered a Mendelian character because *nas 10* nDNA is a nuclear gene locus. This means that these polymorphisms can be also tested for Hardy–Weinberg equilibrium (HWE) and be included in Bayesian clustering multi-genotyping analysis [24, 26], in future multi-locus approaches.

A sufficient sample size (i.e. number of specimens) is required to ensure that the detected fixed differences are maintained even when single specimens from another location are correctly allocated to the correct taxon, i.e. to the three *Anisakis* species. In the present study, we have validated the existence of the fixed differences in a large number of larval and adult specimens of the three *Anisakis* species collected from different fish and cetacean hosts.

The present study has also shown that the combined use of two primer sets for ARMS-PCR, established based on the diagnostic polymorphisms at the *nas 10* nDNA locus, is suitable for direct and rapid genotype determination of the three *Anisakis* species. In particular, primer set-1 discriminated the species *A. simplex* (s. s.) that showed the two fragments at 373 bp and 296 bp, from the species *A. pegreffii*/*A. berlandi,* which have the same banding pattern of 373 bp and 117 bp (Table 3, Fig. 3). Then, the primers set-2 allowed the discrimination between *A. berlandi* with the fragments of 321 bp and 216 bp, *versus A. simplex* (s. s.)/*A. pegreffii* (321 bp, 148 bp). Thus, the use of both primers set is mandatory to discriminate between the three *Anisakis* species (Table 3, Fig. 3).

Tetra-primer ARMS-PCR could be a useful tool for genotyping when direct DNA sequencing could be a time consuming, technically demanding, and costly procedure. For the genetic identification of parasites, such as anisakid nematodes, infecting economically important fish, rapid and cost-effective assays that can be performed with standard PCR instruments are highly desirable. Compared to other genotyping techniques, tetra-primer ARMS-PCR has been reported to be a rapid, reliable, simple and economical assay for SNP genotyping [16, 18, 29, 36]. ARMS can distinguish a single base sequence difference using one-step PCR. This method has recently been developed for SNPs of ITS rDNA for the species identification of *A. pegreffii, A. simplex* (s. s.) and *A. typica* occurring in fish from Korean waters [11]. However, it has also recently been suggested that polymorphic nucleotide positions at these loci of the ITS region of rDNA occur, thus confounding, especially in sympatric areas, the correct identification of the nematodes belonging to the species *A. pegreffii* and *A. simplex* (s. s.) [24]. Thus, in the present study, a tetra-primer ARMS-PCR based methodology was developed based on the novel nuclear marker, for genotyping *A. simplex* (s. l.) nematodes. The assay would be also more convenient than the traditional PCR-RFLP since it eliminates the need for incubation with restriction enzymes. This not only avoids any consequent errors and artefacts from such procedures but also reduces the amount of DNA required for the digestion step in PCR-RFLP. No special equipment and only a small amount of standard PCR reagents are needed in tetra-primer ARMS-PCR. The tetra-primer ARMS-PCR protocol described in the present study is the first reported method enabling genotyping of novel SNPs at the *nas 10* gene locus. This method is rapid, simple, reliable, easy to perform, and economical, and requires a minimum level of expertise. It can be used for both large- and small-scale genotyping studies. The tetra-primer ARMS-PCR technique could also be developed in the future for other nuclear loci that will show diagnostic nucleotide positions between the three species of the *A. simplex* (s. l.) complex.

## Conclusions

The novel nuclear marker (*nas* 10 nDNA) and the development of an ARMS-PCR protocol, based on the diagnostic SNPs detected between the three species of *Anisakis*, proposed in the present study, will allow genotyping of specimens at a further locus when a multilocus-approach is needed. The markers were validated on 219 samples belonging to the three species of *Anisakis* (i.e. *A. pegreffii*, *A. simplex* (s. s.) and *A. berlandi*). Agreement was observed between the identification and genotyping obtained by the tetra-primer ARMS PCR assay and direct DNA sequencing. The protocols detailed here outline methods that can be used to analyze further genomic DNA of the three sibling species of *Anisakis* for two diagnostic mutations.

Currently, SNP markers are one of the preferred genotyping approaches, because they are abundant in the genome, genetically stable and amenable to high throughput automated analysis of *Anisakis simplex* (s. l.) [17]. Diagnostic SNPs in further candidate nuclear genes in the species of *A. simplex* (s. l.) may be useful in multilocus genotyping protocols required for studying micro-evolutionary aspects. This will help to reach a high number of individuals of the three *Anisakis* species genotyped at several nuclear loci, including the novel nuclear locus, DNA microsatellites, and other gene loci [3, 24, 26]. This approach will be useful to our further understanding of hybridization and/or introgression phenomena between the species of the *A. simplex* (s. l.) complex in sympatric areas.

## Conflict of interest

The authors declare that they have no conflict of interest.
